# Over-expression of a Codon Optimized Yeast Cytosolic Pyruvate Carboxylase (PYC2) in CHO Cells for an Augmented Lactate Metabolism

**DOI:** 10.3389/fphar.2017.00463

**Published:** 2017-07-17

**Authors:** Sanjeev K. Gupta, Ankit Sharma, Hiralal Kushwaha, Pratyoosh Shukla

**Affiliations:** ^1^Advanced Biotech Lab, Ipca Laboratories Ltd. Mumbai, India; ^2^Enzyme Technology and Protein Bioinformatics Laboratory, Department of Microbiology, Maharshi Dayanand University Rohtak, India

**Keywords:** monoclonal antibody, super-transfection, codon optimization, yeast pyruvate carboxylase, Chinese hamster ovary cells, stable pool

## Abstract

Monoclonal antibodies are the most demanding biotherapeutic drugs now a days used for the cure of various critical illnesses. Chinese hamster ovary (CHO) cells are one of the main hosts used for the large scale production of these antibodies. However, the cell line and production processes are the key factors to determine the cost and affordability of these antibodies. The metabolic waste lactic acid and ammonium are accumulated during a cell culture process and adversely affects productivity as well as product quality. To control the lactate metabolism of mAb (IgG1-kappa) producing CHO clones, we super-transfected the cells with a mammalian construct bearing codon optimized yeast cytosolic pyruvate carboxylase (PYC2) and a strong fusion promoter for optimal expression of PYC2 enzyme. A pool study was also performed for the assessment of cell’s performance, post-translational modification of a mAb and its expression in a CHO clone. The current study resulted an improved mAb titer up to 5%, galactosylation up to 2.5-folds, mannosylation up to twofold and marginal improved main and basic peaks in the charge variant profile at the cell pool stage. Such, approach may be suitable for the implementation in CHO cells producing recombinant protein for a better process control for the production of biotherapeutics.

## Introduction

Continuous cell lines (CCLs) involve in the accumulation of wasteful byproducts in the form of lactic acid and ammonium which are produced from glucose and glutamine metabolism. Metabolic engineering has been practiced to lower the accumulation of these wasteful byproducts in the culture which results in enhanced product titers as well as product quality which is possible by genetic manipulation of the host cells (Gupta and Shukla, 2016b). Whether to sustain modern therapeutic antibody development or to respond to market demand, the capacity to generate cost effective, high titer and improved quality of a therapeutic recombinant antibody by the biopharmaceutical manufacturers has been remain challenging (Gupta and Shukla, 2015). The development and accomplishment of high yielding fed-batch culture played a great role in enhancing the overall yield of the cell cultures (Bibila and Robinson, 1995; Wurm, 2004; Wlaschin and Hu, 2006). In a fed-batch culture, the extended cell viability is generally linked to the extent of byproducts ammonia and lactate accumulation in the culture medium, which can be harmful to cell growth and product quality (Lim et al., 2010; Slivac et al., 2010). Long-term Chinese hamster ovary (CHO) culture shows a desynchronized metabolism of glucose associated with high lactate accumulation, that can lead to medium acidification followed by undesired increased osmolality due to the addition of alkali for the maintenance of culture pH (Omasa et al., 1991; Ozturk et al., 1992; Lao and Toth, 1997; Cruz et al., 2000). Glycosylation pattern of monoclonal antibodies and other glycoproteins can be altered by the ammonia accumulation (Borys et al., 1994; Andersen and Goochee, 1995; Gawlitzek et al., 2000; Yang and Butler, 2002; Chen and Harcum, 2006).

A number of approaches have been implemented for the reduction of culture byproducts using either process optimization or metabolic engineering. This includes the use of nutrient substitution approaches, for example, replacing the glutamine with asparagine (Kurano et al., 1990) or glutamate (Hassell and Butler, 1990; Altamirano et al., 2000) or substitution of glucose to pyruvate (Genzel et al., 2005) or galactose (Altamirano et al., 2000, 2004, 2006). Accumulation of lactic acid and ammonium during a fed-batch process can also be minimized by maintaining the glutamine and glucose at very low level (Omasa et al., 1992; Zhou et al., 1995). Under depleted nutrients in a culture, the cell metabolism is shifted toward a more proficient metabolic state characterized by a reduced molar ratio of glucose/lactate (Chee Furng Wong et al., 2005; Maranga and Goochee, 2006). The over-expression of glutamine synthetase (GS) gene in CHO cells allows growth in the glutamine free medium, which led to a significant reduction of byproduct ammonia in the culture (Paredes et al., 1999; Zhang et al., 2006). Partial disruption of the gene responsible for the synthesis of lactate dehydrogenase Many researchers over-expressed the cytosolic pyruvate carboxylase 2 (PYC2) gene in different mammalian cell lines, such as BHK cells (Irani et al., 1999) and HEK293 cells (Elias et al., 2003; Henry and Durocher, 2011; Vallée et al., 2014) which has shown a significant reduced lactate accumulation.

Irani et al. (2002) engineered the BHK-21 cells with yeast pyruvate carboxylase in the cytoplasm to increase the glucose flux into the TCA to improve the ATP content. The cells over-expressing PYC2 gene showed twofold higher glucose consumption and twofold increased erythropoietin yield in a bioreactor culture as compared to the control sample. Fogolín et al. (2004) investigated the impact of temperature shift and the PYC2 expression on the CHO cells capable of expressing recombinant human granulocyte macrophage colony stimulating factor (rhGM-CSF). The cell expressing PYC2 showed a poor cell growth and inferior peak cell concentration in contrast to the control cells expressing rhGM-CSF. But, the production of lactate was down-regulated up to 65% and specific productivity of protein-enhanced by 200% as compared to the control sample. Kim and Lee (2007) introduced the human pyruvate carboxylase (hPC) to analyze its effect on lactate formation in DG44 cells. The lactate production rate was diminished by 21–39% as compared to the control cell line. Henry and Durocher (2011) also found the enhanced glycoprotein production in the HEK-293 cells over-expressing the pyruvate carboxylase gene.

In another approach, the amplification of hPC gene in CHO-DG44 cells resulted in a minor reduction in the lactate accumulation (Kim and Lee, 2007). Fogolín et al. (2004) over-expressed the PYC2 in CHO cells, which leads to decreased specific lactate production, but the molar ratio of lactate/glucose remained high (∼1.5). Wilkens and Gerdtzen (2011), analyzed that lactate production was originated through pyruvate increase due to its improved synthesis rate in the glycolytic pathway and restricted consumption in the TCA cycle, which promotes the lactate production.

In recent times, over-expression of PYC2 in CHO cells was found to lengthen the culture durability and decrease lactate glucose ratio by 25%, but at the cost of a 50% reduction in the cell specific antibody production rate (Wilkens and Gerdtzen, 2015). Toussaint et al. (2016) over-expressed the yeast pyruvate carboxylase (PYC2) gene into CHO cells expressing a monoclonal antibody used against epidermal growth factor receptor (EGFR) and observed an increased titer in both shake flask (batch and fed-batch) as well as bioreactor culture. In a bioreactor culture (fed-batch mode), the PYC2 over-expression resulted into a net gain of 20% in the final volumetric productivity.

In the current study, we used codon optimized yeast pyruvate carboxylase gene for the over-expression in a CHO-derived mAb clone (IgG1-kappa antibody) for improved cell growth, product titer and it’s quality. Further, we also investigated the potential role of the PYC2 over-expression on the pyruvate and lactate metabolism of a CHO clone producing mAb at the pool stage. A higher concentration of selection agent was used for stable pool selection and its metabolic study. Furthermore, the PYC2 engineered mAb clone was also assessed for the impact on the product quality attributes such as *N*-glycosylation pattern and charge variant distribution of a therapeutic mAb.

## Materials and Methods

### Codon Optimization and Gene Synthesis

The gene encoding yeast cytosolic pyruvate carboxylase (PYC2) was chemically synthesized and codon optimized for CHO cells obtained from GeneArt, Germany (Life Technologies/Thermo, United States). During gene design, appropriate restriction sites were incorporated at either end of the synthetic gene to allow cloning of PYC2 gene at multiple cloning sites (MCS) of selected mammalian expression vector.

The synthetic gene was synthesized with 62% GC content and 0.97 codon adaptation index (CAI) (Supplementary Figure 1). The codon usage was adapted to the codon bias of organism *Cricetulus griseus* genes (CHO). In addition, regions of very high (>80%) or very low (<30%) GC content has been avoided where possible. During the optimization process the *cis*-acting sequence motifs such as internal TATA-boxes, chi-sites and ribosomal entry sites, AT rich or GC-rich sequences, RNA instability motifs, repeat sequences and RNA instability motifs and splice donor and acceptor sites in higher eukaryotes were avoided where applicable.

The gene was reconstituted and confirmed by agarose gel electrophoresis and was found intact (no degradation). The same was further used for *E. coli* DH5α transformation. The miniprep DNA isolated from randomly selected colonies was confirmed by digestion with EcoR*V* and Xho*I* restriction enzymes (Supplementary Figures 2A,B).

### PYC2 Cloning in a Mammalian Expression Vector

A mammalian expression vector pCHO_11 (vector backbone used from Invitrogen/Thermo Fisher, United States) bearing dual resistance gene marker was used for the cloning of the cytosolic PYC2 gene for metabolic engineering of the CHO cell line. The PYC2 gene bearing vector was named as pMPYC (**Table 1**). The pMPYC vector contains a kanamycin resistance marker for the clone selection in *E. coli* whereas Puromycin and DHFR (Di-hydrofolate reductase) markers are used as a dual selection marker for the stringent phenotypic selection of stably transfected CHO cells in the presence of various concentrations of Puromycin and MTX (Methotrexate).

**Table 1:** Description of the hosts and vectors used for the PYC2 engineering.

Sr. No.	Host/Vector	Characteristics	Purpose
1.	pCHO_11 (expression vector)	Posses Kanamycin, Puromycin and DHFR markers	For cloning, clone selection and PYC2 expression
2.	pMPYC	pCHO_11 vector containing PYC gene	For transfection and PYC2 over expression in CHO
3.	*E. coli* DH5α	Compatible for pCHO_11 plasmid	For the cloning in *E. coli* (pMPYC vector construction)
4.	CHO-S	Mammalian expression host (suspension cell line)	For transfection and overexpression of PYC2 gene (Metabolic study)

Codon optimized PYC2 gene fragment was cloned at Avr*II* and BstZ*II71* cloning sites (MCS) of the pCHO_11 vector. The same enzymes (Thermo Fisher, United States) were used for the clone verification. The DNA sequence of cloned gene was verified by DNA sequencing for the construct confirmation. For the preparation of DNA in large amount to be used for CHO transfection, GeneJet plasmid extraction Midi-prep kit was used from Thermo Fisher, United States.

### Cell Line, Medium and Feed

The CHO-S a suspension cell line (Gibco/Thermo Fisher, United States) is used as a production host for mAb expression studies as well as stable clone development which was then ultimately used for PYC2 modulation to study the effect of lactate metabolism. The chemically defined and animal component free growth medium CD-CHO (Gibco/Thermo Fisher, United States) was used for CHO-mAb clone transfection, stable pool generation, and shake flask studies. The cell growth medium is supplemented with 6–8 mM L-glutamine (Gibco/Thermo Fisher, United States), and 1× anti-clumping agent (ACA) (Gibco/Thermo Fisher, United States). Various concentration of the selection agents, Puromycin 10 mg/mL (Gibco/Thermo Fisher, United States), and Methotrexate (MTX) (Sigma–Aldrich, United States) were used for the stable pool selection. The cell culture experiments were carried out in a CO_2_ incubator set at 37°C, 80% humidity and 8% CO_2._ The Feed A and B (Hyclone/GE, United States-United Kingdom) were used as a supplement for the shake flask fed-batch studies. The D-Glucose (Sigma, United States) was used as a carbon source for cell culture experiment.

### Super-Transfection of CHO-mAb Clone with PYC2 Plasmid

To study the effect of PYC2 on CHO expressing mAb gene (IgG1-Kappa, the clinical indication and specificity of the target antigen is not disclosed due to its confidentiallity) to, CHO clone expressing mAb generated in house (30 μg/mL puromycin and 500 nM MTX) was transfected with the pMPYC construct bearing the gene yeast pyruvate carboxylase (PYC2). CHO-mAb cells were transfected with the pMPYC construct using Neon electroporator (Neon^®^ Transfection System, Invitrogen/Thermo Fischer, United States).

Prior to transfection, the purified plasmid DNA was linearized with restriction enzyme Nru*I* for a successful transfection exercise. The cells were cultured at 4.0 × 10^7^ viable cells/10mL in a T75 flask for 25 μg plasmid transfection. We performed electroporation of CHO-mAb clone with the linear pMPYC plasmid at 1550 V for 10 ms and with 3 pulses.

Forty-eight hours of post transfection, cells were subjected for the pool generation by using 50 and 75 μg/mL Puromycin and 1000 and 2000 nM MTX (Higher concentration than a CHO-mAb clone). The fed-batch study was performed in 30 mL complete CD-CHO medium +6 mM glutamine in a sterile 125 mL shake flask. The CHO-mAb clone (without PYC2) and CHO-mAb-PYC2 pools were cultured and used for the shake flask studies.

For a fed-batch shake flask study, the selected cell pools as well as parental CHO cells were seeded with 0.5 × 10^6^ cells/mL into 30 ml CD-CHO growth medium (Gibco/Thermo Fisher, United States) supplemented with 6 mM glutamine and 50 μg/ml dextran sulfate (ACA) in a 125 ml shake flask and incubated at 37°C, 8% CO_2_ at constant agitation of 140 rpm in a CO_2_ shaker incubator (Kuhner, AG). The feed supplement feed A 1.3% and feed B 0.13% (Hyclone/GE-United States-United Kingdom) were fed to the culture from day 3 onwards and maintained according to the manufacturer’s instruction. A high concentration 8 g/L (44.5 mM) of glucose was maintained through day 3 to batch end. The cultures were monitored everyday by taking 0.5–1.0 mL cell culture aliquot for the cell growth and metabolic studies.

### PYC2 Expression Assay by ELISA

The PYC2 protein expression obtained from the selected pool and mAb clone was analyzed using Enzyme-linked Immunosorbent Assay (ELISA). The protein assay was performed from the cell extract isolated from selected pools. A polyclonal goat-anti-PYC antibody (Sigma, United States) was used for the capture of PYC2 protein, whereas secondary antibody anti-goat-HRP conjugated (Sigma, United States) for detection of the protein. The PYC expression was determined using OD value taken at 450 nm as the protein concentration couldn’t be measured due to unavailability of the commercial PYC protein for the standard curve generation. The cells were collected from each pool and washed with 1× PBS (phosphate saline buffer) buffer and lysed for the protein extraction and ELISA assay.

### Cell Growth and Spent Medium Analysis

The cell count and viability of the shake flask culture were analyzed using trypan blue in a VI-cell counter (Beckman Coulter Inc., Fullerton, CA, United States). The glucose and lactate concentrations were determined using YSI biochemical analyzer Model 2700 analyzer (Yellow Springs Instruments, United States). The mAb concentration was measured using high-performance liquid chromatography (HPLC, Waters, United States) Protein-A affinity column (Analytical column). The protein samples were collected at different time intervals to analyze the mAb titer by HPLC method.

### Glycan Analysis

Culture supernatant generated from the shake flask was purified by Protein-A affinity chromatography and further used for glycan and charge variant analysis. An Acquity UPLC HILIC-BEH Glycan chromatography column (1.7 μm, 2.1 mm × 150 mm) was used for glycan analysis in Acquity UPLC H class (Waters, United States) system. The eluent mobile phase-A (50 mM ammonium formate, pH 4.5) and mobile phase-B (Acetonitrile) were used to run the HPLC for glycan analysis. The peaks obtained were detected using Fluorescence detector (λ ex = 330 nm and λ e = 420 nm). For analysis, Protein A purified samples (20 μl) were denatured and subsequently deglycosylated using PNGaseF enzyme (NEB, United States) and incubated at 37°C for 3 h. The deglycosylated samples were dried by speed vacuum concentrator (Thermo Fisher Scientific, United States) and then released N-Linked Glycans were labeled with 2-AB reagent (Waters, United States) by incubating in a heating block at 65°C for around 2.5 h. The dried sample was reconstituted in 50 μl of Mobile Phase A and B buffers and injected 10 μl on HILIC-UPLC BEH Glycan column for the separation of N-linked glycans. The peaks obtained were analyzed using Empower 3.0 software.

### Charge Variant Analysis

The Alliance HPLC (Waters, United States) system was used for the mAb charge distribution analysis. The chromatography column TSK gel CM-STAT (4.6 mm ID × 100 mm L, 7 μm), Tosoh was used with the flow rate of 0.8 mL/min, column temperature 30°C and UV detection at 280 nm. The eluent derived were used for the charge variant HPLC run. Hundred microliter of Protein-A purified sample was injected on CEX-HPLC column. Acidic, main and basic peaks were resolved between 8 and 30 min. The peaks obtained were integrated for area % determination using Waters Empower 3.0 software.

## Results

To enhance the cellular metabolic activity and improve the cytosolic and mitochondrial metabolic network, we modulated the CHO-mAb cell’s pyruvate flux by super-transfection with PYC2 gene. In the current study, the codon optimized yeast pyruvate carboxylase (PYC2) gene was used for the cloning and super-transfection of CHO-mAb clone, which triggers the cytosolic pyruvate toward the TCA cycle of the cell’s metabolic pathway.

### Clone Development for Codon Optimized PYC2

The synthetic gene obtained from GENEART (Germany) was codon optimized for CHO cells and synthesized and cloned into pCHO_11 vector at BstZ17*I* and Avr*II* restriction sites.

**FIGURE 1:**
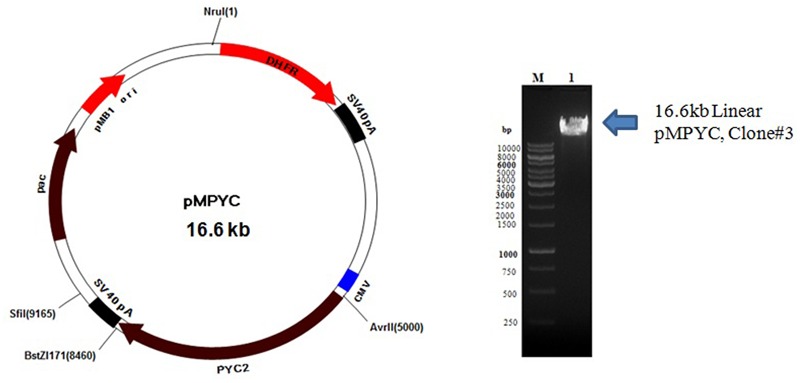
Linearization of pMPYC construct with Nru*I* enzyme for CHO transfection. Lane M, 1 kb DNA ladder; Lane 1, Linear DNA, pMPYC, Clone #3 (∼16.6 kb).

Colonies obtained from the *E. coli* transformants were confirmed by miniprep DNA isolation followed by restriction digestion with BstZ17*I* and Avr*II* enzymes. The expected size of DNA fragment (∼3.6 kb) was released from pCHO_11_PYC2 construct which confirms a successful cloning of PYC2 gene into a pCHO_11 vector. Out of three clones screened, two clones, clone #1 and 3 were found positive (**Figure 1**). The pCHO_11_PYC2, clone# 3 (named as pMPYC) was sequenced using plasmid and gene specific primer and found 100% match with the original sequence (codon optimized). The same clone was further used for transfection and CHO cell engineering.

For CHO transfection, the midiprep DNA (450 ng/μl with 260/280 ratio of 1.945) of positive clone pMPYC was subjected for linearization with Nru*I* enzyme and a linear DNA was observed of desired size (∼16.6 kb) on the agarose gel (**Figure 1**). The linearization of DNA may not improve the transfection efficiency, but, it allows efficient DNA integration into the host genome without disrupting the gene of interest and other elements present in the vector required for expression in the mammalian host.

To enhance the energy metabolism and pyruvate flux toward TCA cycle, the CHO-mAb clone is engineered for the metabolic benefits. The CHO-mAb clone was transfected with the plasmid pMPYC containing PYC2 gene by using neon electroporation as described in material and methods. To select a stable cell pool, from the CHO-mAb clone, we applied a higher concentration of selection agents than was used for pool selection of pCHO-mAb clone (P30/M500). In this scheme, we generated the transfected CHO cell population using 50 μg and 75 μg/mL, puromycin and 1000 and 2000 nM MTX, respectively. But, the pool subjected with 75 μg/mL puromycin and 2000 nM MTX couldn’t recovered may be due to high concentration of the selection agents where as the cell pool selected at 50 μg/mL puromycin and 1000 nM MTX recovered well and used for the future study. The selected cell pool (P50/M1000) may have a mix population of transfected PYC2-CHO cells harboring the varying number of the PYC2 gene copies also with a changing PYC2 expression pattern due to the random integration event as well as position effect in the CHO genome.

### PYC2 Expression Study by ELISA

**FIGURE 2:**
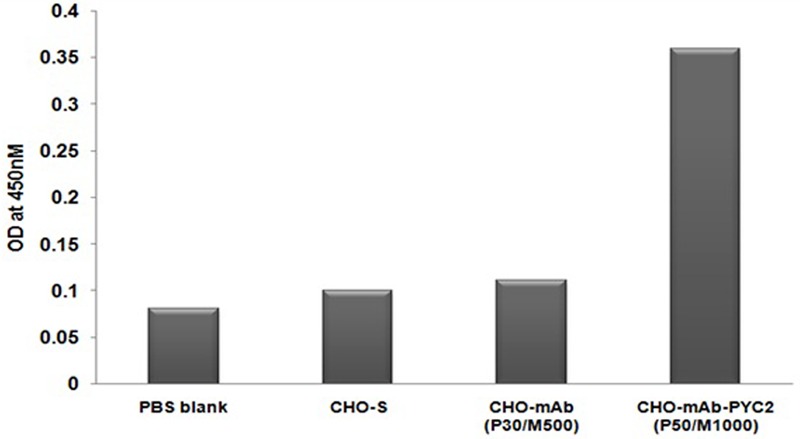
Enzyme-linked Immunosorbent Assay for PYC2 expression study from the cell extract of the CHO-mAb and CHO-mAb-PYC2 cells.

To check the protein expression of PYC2 gene, the protein is extracted from the well recovered selected cell pool and control cell and subsequently, performed ELISA assay using the goat-anti-PYC2 antibody as described in material and methods. The cell number was normalized for each pool and protein was extracted after the disintegration of the cells collected from different pool. Further, anti-goat HRP conjugated antibody was used as a detection antibody. The protein expression of stable pool (CHO-mAb-PYC2 selected at P50/M1000) was compared with the CHO-mAb parental clone (P30/M500). A relative protein quantitation is performed by comparing the OD of control CHO-mAb cells obtained at 450 nm. The amount of protein observed was 3- to 4-folds higher (**Figure 2**) than that of the control cells. The selected pool generated was taken up for the shake flask pool study.

### Cell Metabolic Profile

**FIGURE 3:**
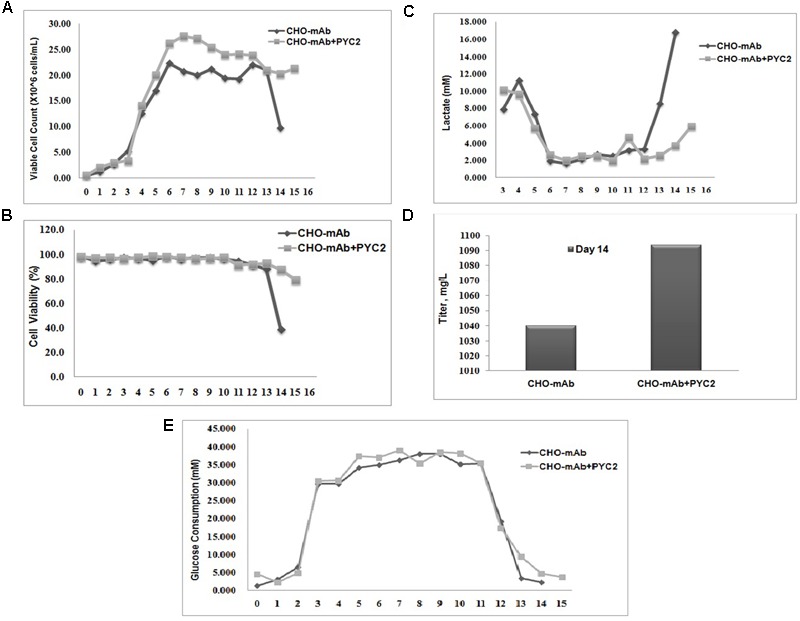
Comparative fed-batch study of a mAb clone engineered with PYC2 gene and a set of control without PYC2 over-expression: **(A)** Cell density, **(B)** Cell viability, **(C)** Lactate profile, **(D)** Titer profile and **(E)** Glucose profile.

To evaluate the metabolic behavior of the selected cell pool (P50/M1000) and its efficiency toward central carbon metabolism, the stoichiometric ratio between lactate production and glucose consumption (Δ*L/*Δ*G*) has been studied in comparison with the control cells. The lactate consumption rate of the CHO-mAb-PYC2 pool was approximately fourfolds greater (day 14) as compared to the control cells (**Figure 3**). The Δ*L/*Δ*G* ratio of both control and PYC2 modulated cells were calculated, and were 0.236 and 0.008, respectively. The above results indicated that the PYC2 modulated cell pool have more efficient central carbon metabolism than the one exhibited by control cells.

### PYC2 Over-expression and Its Effect on mAb Expressing Clone

Chinese hamster ovary clone producing monoclonal antibody requires the production of high titer and high-quality non-immunogenic antibody with desired *N*-glycosylation and charge distribution profiles. The glycosylation composition of a mAb shows a major impact on the efficacy and safety of a therapeutic drug and the product quality attribute is maintained by controlling the process parameters during bio-pharmaceutical production. In this study, we investigated the impact of the PYC2 super-transfection on the cell culture profile, titer, *N*-glycosylation and charge variant profiles of a mAb. In order to understand the potential role of the optimized PYC2 in CHO cellular machinery, we transfected the pMPYC2 construct in CHO cells which is stably expressing a therapeutic monoclonal antibody (mAb) and selected the pool derived from 50 μg/mL Puromycin and 1000 nM MTX selection agents. We, further scaled-up the transfected pool in a shake flask for a fed-batch cell culture study. We observed that the cell density of PYC2 modulated pool showed ∼5 million/mL higher and extended cell viability more than 80% till day 15 as compared to the control cell (**Figures 3A,B**). We also observed a mark reduction (about 4- to 5-folds) in the lactate accumulation as compared to the control cell, however, glucose consumption profile was similar in both the cultures (**Figures 3C,E**). Expression of the antibody in fed-batch culture with PYC2 modulation was not increased significantly. However, there was a marginal increase of approximately 5% titer observed on day 14 as compared to the control cells during the exponential phase of the cell growth (**Figure 3D**). The increased product titer was obtained at heterogeneous pool stage which may become 2–5 times more after single cell dilution cloning, clone selection and process optimization. It can be assumed that the, improved cell density and extended cell viability of the culture will definitely add value in terms of controlling the cell culture process and achieving a better product quality at higher scale in comparison to the control cell. Further, the product quality attributes of the mAb is assessed from both control as well as experimental pool to assess the effect of controlled lactate metabolism of PYC2 modulated cell pool.

### Effect of PYC2 Engineering on mAb Quality

**FIGURE 4:**
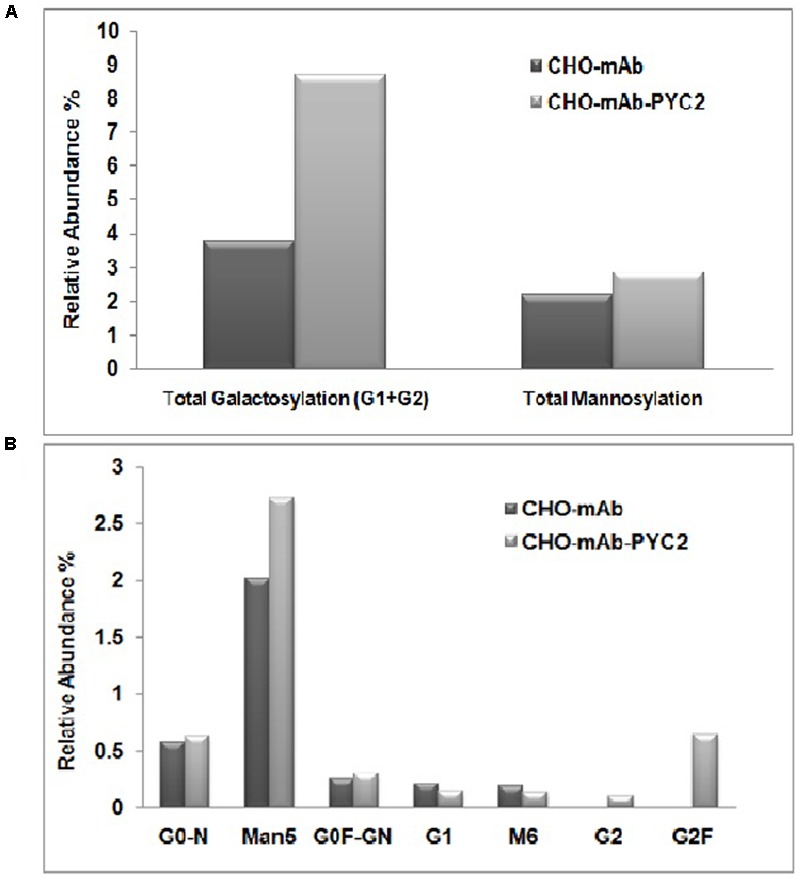
Comparative glycoform analysis of a mAb secreted from the engineered PYC2 cells and a set of control without PYC2 over-expression. **(A)** Total relative abundance of the galactosylation and mannosylation. **(B)** Relative abundance of glycan composition.

To assess the mAb quality attributes, we collected the samples from the grown culture for both control and PYC2 modulated pool and further analyzed charge heterogeneity as well as *N*-glycosylation pattern of the expressed mAb. The glycosylation pattern of any mAb plays a pivotal role in CDC (complement-dependent cytotoxicity) activity and is responsible for the enhanced efficacy of a molecule. We investigated the impact of PYC2 on various glycosylation distributions which is critical for the development of an active and potential mAb. The result obtained showed a significant difference in the glycan composition of mAb obtained from both the cultures. We observed a total improvement in galactosylation composition by approximately 2.5-folds, in contrast a marginal increase (∼1.0%) in the mannose composition was also observed in PYC2 modulated pool (**Figures 4A,B**). The low-mannose content is beneficial as it reduces the early blood clearance when administered to the human body, whereas high galactosylation allowed increased CDC activity of a mAb. Relative abundance of glycan pattern of G_0_F-GN, G1 and G2F were found comparatively 3–8% higher with PYC2 modulated mAb producing CHO pool, which is beneficial for *in vivo* potency when used for the treatment of a patient.

**FIGURE 5:**
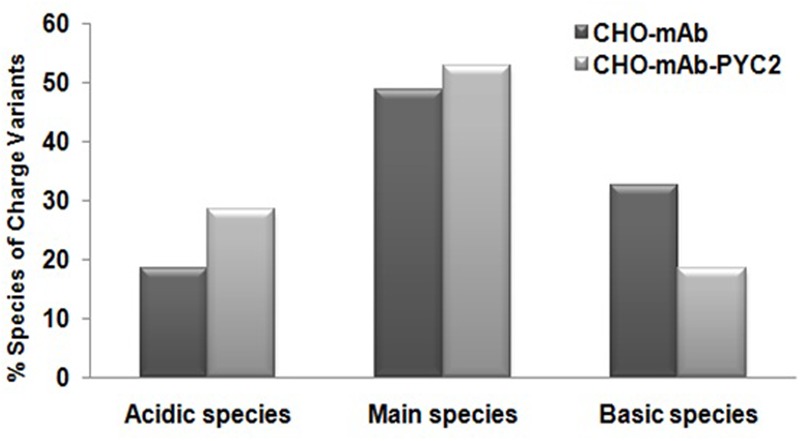
Comparative charge variant distribution analysis of mAb secreted from the engineered PYC2 cells and a set of control without PYC2 over-expression.

In addition, we also assessed the impact of PYC2 over-expression on the charge heterogeneity of the mAb analyzed by CEX-HPLC (cation exchange chromatography), which suggest that over-expression of PYC2 allowed marginal improvement on the main peak (∼5%) but, a significant reduction approximately 15% of the basic peak in the engineered cells compared to the control cells. In contrast, increased acidic peak distribution about 10% was observed with PYC2 engineered sample (**Figure 5**). The improvement in the basic and main peaks distribution observed in the experimental sample will allow overall increased protein recovery in the purification process, thus allows cost effective process development.

## Discussion

The most demanding mammalian cell lines CHO cells have become an industry standard expression host for the production of recombinant therapeutic proteins represents a huge impact on the biopharmaceutical industry (Gupta and Shukla, 2015, 2016a). The mammalian CHO cells are potentially used for such applications due to their ability to appropriately assemble and secrete functional proteins for the use of critical illness. These mammalian production systems show a metabolic behavior linked with a high cell proliferation rate, which involves a high consumption of glucose and glutamine, resulting in the accumulation of cell culture waste products ammonium and lactate. The waste accumulation potentially contributes to the limitation of cell growth, a shift in the culture pH toward acidic, triggers early viability drop through apoptotic processes and thus reduced product titer and poor quality of a recombinant protein (Lao and Toth, 1997; Wagner et al., 1998; Gambhir et al., 2003; Altamirano et al., 2013). It is studied that, the lactate concentration lower than 20 mM does not affect cell growth or productivity, however, if it is accumulated in the range of 20–40 mM impair the productivity and over 40 mM inhibits cell growth (Wagner et al., 1998).

In the present study, we engineered a mAb producing CHO cell line with PYC2 gene to control the lactate metabolism of the cells, which resulted into higher cell density, extended batch duration and a well controlled central carbon metabolism. It is studied that, the expression efficiency of endogenous pyruvate carboxylase gene (PYC2) is slower in CHO cells and a high rate of glycolysis during cell culture process leads to rapid pyruvate accumulation in the cytoplasm which poorly shifts toward mitochondrial metabolic network (Jitrapakdee and Wallace, 1999). Although, yeast pyruvate carboxylase gene is engineered to tune up the cellular metabolic activity in various mammalian cell lines (CHO, BHK21and HEK2) which were used as a host for the expression of many recombinant products (Huang et al., 2010; Henry and Durocher, 2011; Wilkens and Gerdtzen, 2015), however, the current study was done to see the effect of PYC2 engineering on mAb quality attributes at cell pool stage in controlled lactic acid metabolism. In engineered BHK21 cells, achieved high cell density, had an extended lifespan and also exhibited a more efficient metabolism producing less lactate per glucose consumed (Irani et al., 2002). In another study done by Fogolín et al. (2001, 2004), PYC2 was over-expressed in the CHO cells which showed a drop in cell density, but at the same time cells exhibited improved lifespan and cellular metabolism. Several attempts have been made so far for the improvement of culture sustainability, productivity and quality of the recombinant protein by altering the metabolic behavior of the established cell lines by using either process optimization or host cell engineering. However, success mainly depends on the host cell, the nature of the recombinant protein and the conditions of the cell culture processes (Altamirano et al., 2013).

### PYC2 Codon Optimization for Optimal Expression

In the current study, we used a codon optimized yeast pyruvate carboxylase gene for an optimal PYC2 gene expression in CHO cells which can lead to enhanced metabolic flux of the cytoplasm and mitochondria. The codon optimized genes are extensively used for expression studies and the production of heterologous proteins by academia as well as the biotechnology industries (Rosano and Ceccarelli, 2014). The codon optimization allows enhanced volumetric productivity of the recombinant proteins and metabolites, thus leads to a cost effective process and product development. Besides, this also impacts the economic feasibility of biotechnological process, significantly (Elena et al., 2014).

Here in the current study, we used codon optimized yeast cytosolic pyruvate carboxylase, the optimized gene should therefore allow high and stable expression rates in CHO cell line. This approach was used to minimize the first rate limiting step of a cell culture process.

### CHO Metabolic Engineering with PYC2

To develop a metabolically efficient CHO cells for controlled lactic acid metabolism in a cell culture process, the codon optimized PYC2 gene was cloned into a mammalian expression vector bearing two selection marker genes as well as a strong promoter with the expectation of an efficient modulation of PYC for controlled metabolism. From the PYC2 transfected heterogeneous pool, we applied a higher concentration of selection agents MTX and puromycin (P50 μg/mL and 1000 nM of MTX-P50/M1000) than that of a mAb clone selected at M500 and P30 concentrations. The above strategy was adapted to ensure a stable integration of PYC2 gene at cell pool stage. The current study reveals that how PYC2 engineering allows an extended cell viability, high cell density and lower lactate production in comparison to the control cells. We presumed such behavior of the cells observed mainly due to PYC2 expression in a mAb producing CHO cell line.

The PYC2 expression assessment in the cell pool reveals stable gene integration in a mAb producing CHO cell line. The pool study was also carried out in comparison with the control cell line which showed a significant difference in the overall cell culture performance as compared to the PYC2 transfected pool.

The cell pool engineered with PYC2 gene and selected at higher concentration of selection agents has shown a peak cell density of ∼28 × 10^6^ cells/mL and sustained culture till day 15 with 80% cell viability in comparison to the control cells, which has shown the peak cell density of ∼23 × 10^6^ cells/mL with 40% viability, on day 14. The PYC2 engineered cells have shown ∼5 million higher cell count and 3 days extended cell viability as compared to the control cells. The difference in the cell count and culture longevity is indicative of the differences in their metabolic behavior caused due to controlled lactate metabolism in PYC2 engineered cell pool. In terms of lactic acid metabolism, a significantly higher consumption is observed in PYC2 engineered pool (below 3 mM) during exponential and stationary phase, whereas in control cells, there was a metabolic switch from lactate consumption to accumulation observed between 11 and 12 days during the stationary phase of the culture, which has gone up from 3 to 17 mM between 12 and 14 days. The lactic acid concentration gone beyond 15 mM in the control cells is may be due to uncontrolled lactic acid metabolism. The surged lactic acid in the control cells has led to early and a steep viability drop after day 12 of the cell culture process, which is detrimental for protein production as well as its quality in terms of glycosylation profile (Wagner et al., 1998; Altamirano et al., 2013).

In a previous study, it has been reported that the over-expression of the yeast pyruvate carboxylase in CHO cells enhances the central carbon metabolism and exhibited maximum cell density around 12–14 million cells/mL and viability has been close to 80% on day12 (Fogolín et al., 2001; Zhou et al., 2011; Vallée et al., 2014; Toussaint et al., 2016). Whereas in the current study, we were able to achieve a cell density of up to 28 million/mL and extended viability up to 80% till day 15 as compared to the control cells which showed 23 million cells/mL and a steep viability drop on day 12. This variation in the cells behavior and metabolic profile of the PYC2 engineered pool attributed may be due to super-transfection of mAb producing CHO cells and increased gene load of the PYC2 gene. In terms of protein expression profile, at the cell pool stage, we observed an enhanced marginal expression of a mAb up to 5% as compared to the control cells. A marginal increase of mAb titer in the super-transfected cell pool could be because of two reasons, firstly the selected pool have the heterogeneous cell population with an un-optimized PYC2 gene copy load and the low producer clones may have overgrown to the medium and high producer clones and secondly, the cells growing with higher cell density may require more nutrients for the sufficient protein expression. Although, we supplied same quantity of feed supplements to both the cultures.

### Glucose and Lactate Flux Analysis

In a fed-batch shake flask study, a significant reduction of Δ*L/*Δ*G* ratio was observed in PYC2 engineered cell pool as compared to the control cells. Both the cultures have shown a different metabolic state is represented by different lactate to glucose stoichiometric ratios of Δ*L/*Δ*G*. The Δ*L/*Δ*G* ratio of PYC2 engineered pool was 0.008, in contrast, the parental cell line showed significantly higher Δ*L/*Δ*G* ratio, which is 0.236. The above results suggest that PYC2 engineered pool is capable of controlling the lactic acid metabolism better as compared to the control cells.

Wilkens and Gerdtzen (2015) reported a reduced mAb titer when it was expressed in PYC2 engineered cell line. Our study done on PYC2 modulated cell line strongly supports the data published by Toussaint et al. (2016). Earlier studies also revealed enhanced recombinant protein production when expressed in a PYC2 engineered cell line (Irani et al., 2002; Fogolín et al., 2004). Thus, the PYC2 engineering approach may be a beneficial approach for a better cell line, process and product development.

### Effect on mAb’s Quality Attributes

In addition to cell performance and titer study of PYC2 engineered pool, we also explored the impact of PYC2 expression and lactate metabolism on the quality of the recombinant mAb. The production of a recombinant therapeutic monoclonal antibody with desired glycan composition has been a considerable challenge for the bio-pharmaceutical industry (Zhu, 2012). Various successful attempts have been made toward achieving the desired glycosylation pattern of the recombinant proteins shown by cell culture media and feed optimization and use of various heavy metals (Harrison and Jarvis, 2006; LeFloch et al., 2006; Stork et al., 2008). In a production host, the *N*-glycosylation profile is known to be mostly dependent on glycosylation processing enzymes present in the host, the availability of intracellular nucleotide sugar substrates and metabolic activity of the cellular system. In the current study, we performed a fed-batch shake flask study for both control cells, and PYC2 engineered cell pool to assess both titer as well as product quality profile.

In terms of product quality attributes, we analyzed the glycan as well as charge variants of the mAb from the supernatant of both control cell as well as PYC2 engineered cell pool. Wherein, we observed approximately 2.5-fold increased total galactosylation composition of a mAb in an engineered cell pool as compared to the control cells. The increased galactosylation thus undoubtedly will reduce an extra effort required for upstream process optimization for the quality improvement. An efficient and optimized glycosylation of a mAb also allows an increased product efficacy (Jefferis et al., 1998). Another important glycan composition is the terminal mannose composition which we studied in both the cultures. Terminal mannose (Man) residues that have high affinity with various types of receptors in the human affects the blood serum clearance rate of a therapeutic protein (Goetze et al., 2011). In the present study, we observed a marginal increase in the mannosylation of monoclonal antibody in PYC2 engineered pool as compared to the control cells. There was no significant difference in the charge heterogeneity of the molecule obtained in an engineered cell pool when compared with the control cell line. However, there is a marginal improved basic and main peak was observed in the PYC2 engineered cell pool. The above study reveals that the controlled lactate metabolism of PYC2 engineered cells may have significantly led to an improved galactosylation profile of a recombinant mAb.

In the present study, we report the PYC2 engineering of a most demanding host CHO-S cell line and consequence of PYC2 over-expression on the alteration of lactate metabolism, cell growth, therapeutic monoclonal antibody production and its quality attributes. We report the use of codon optimized PYC2 gene for the CHO engineering first time, which has allowed the development of an efficiently engineered cell pool with well-controlled lactate metabolism. The PYC2 modulated cell pool, generated using a higher concentration of selection agent have shown an active metabolic switch from lactate accumulation toward lactate consumption, increased cell density, improved viability, prolonged culture, increased volumetric productivity and improved glycosylation profile as compared to the control cells. The assessment of mAb quality attributes such as glycosylation and charge variant distribution indicates how PYC2 over-expression helped in the quality improvement when compared with the control cells, which may help in improving the *in vivo* potency of a therapeutic recombinant product (mAb).

## Conclusion

The current study reveals that an optimal expression of the PYC2 gene in CHO cells enhances its metabolic activity, which in turn directly or indirectly affects the overall cell’s performance, including titer and glycosylation composition (post-translational modification) of a therapeutic mAb. We also demonstrated that the PYC2 engineering in CHO cells could be helpful in improving the titer and quality of a recombinant therapeutic protein significantly by tuning the CHO metabolic pathway. Further, PYC2 engineered cell pool that has shown better performance compared to the control cell can be used for clone development and as an expression platform for cell line development and well controlled upstream process in bioreactor for the production of an affordable therapeutic recombinant protein. Based on the current study, we conclude that the PYC2 modified cell line can potentially be used as an expression platform for an improved protein titer with desired product quality when used for expression of the therapeutic recombinant proteins.

## Author Contributions

SG and PS are designed the experiments. SG and AS conceived and performed most of the experiments. HK performed the analytical tests for protein titer and quality assessment. SG and PS wrote the manuscript.

## Conflict of Interest Statement

The authors declare that the research was conducted in the absence of any commercial or financial relationships that could be construed as a potential conflict of interest.
