# Supplementary material for: Over-expression of a Codon Optimized Yeast Cytosolic Pyruvate Carboxylase (PYC2) in CHO Cells for an Augmented Lactate Metabolism
Source: Front Pharmacol. 2017 Jul 17;8:463. doi: 10.3389/fphar.2017.00463 (PMC5511841; doi:10.3389/fphar.2017.00463)
Supplement: Supplementary file 1 [file Presentation_1.PPT]

## Slide 1
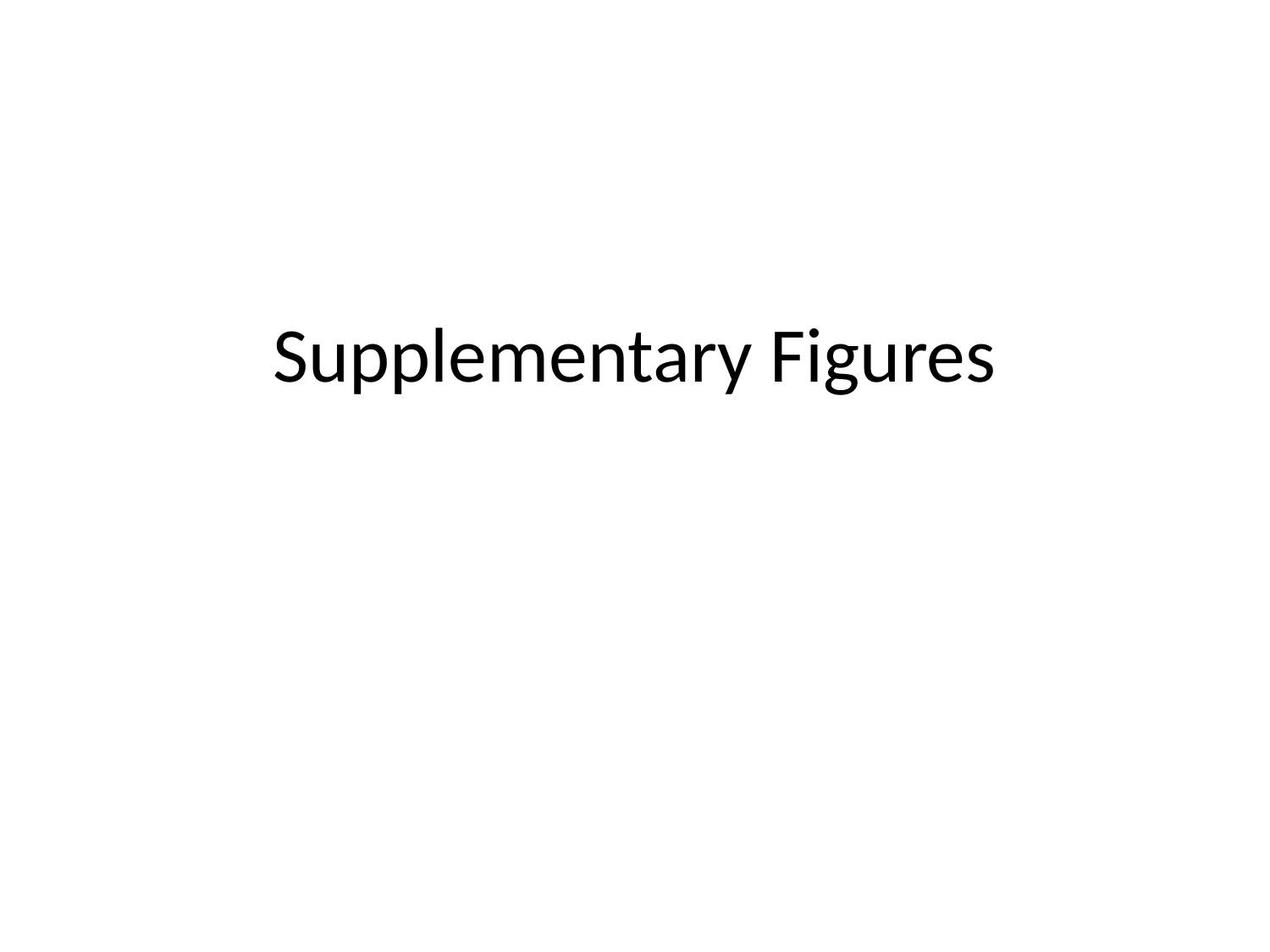

# Supplementary Figures

## Slide 2
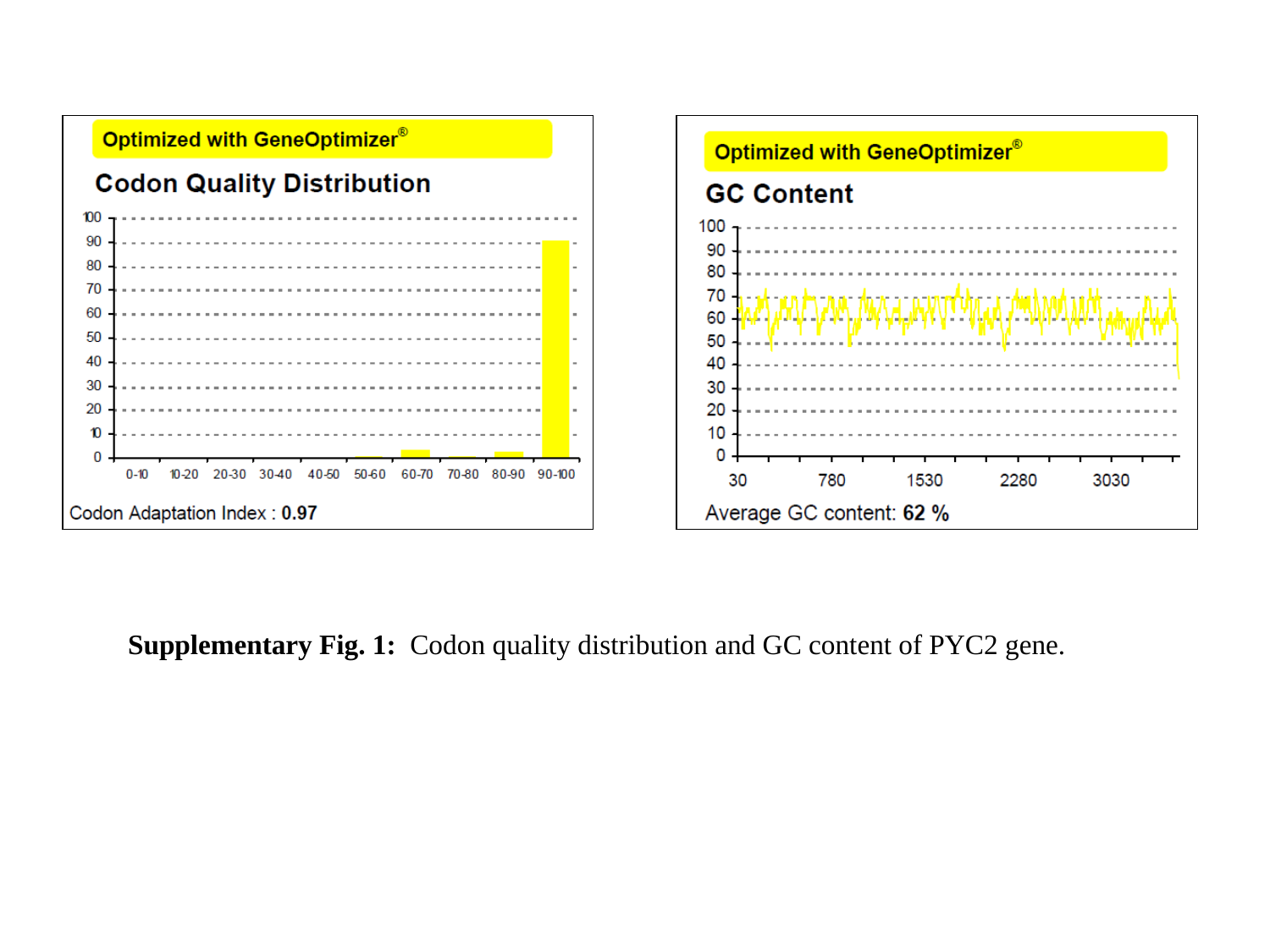

Supplementary Fig. 1: Codon quality distribution and GC content of PYC2 gene.

## Slide 3
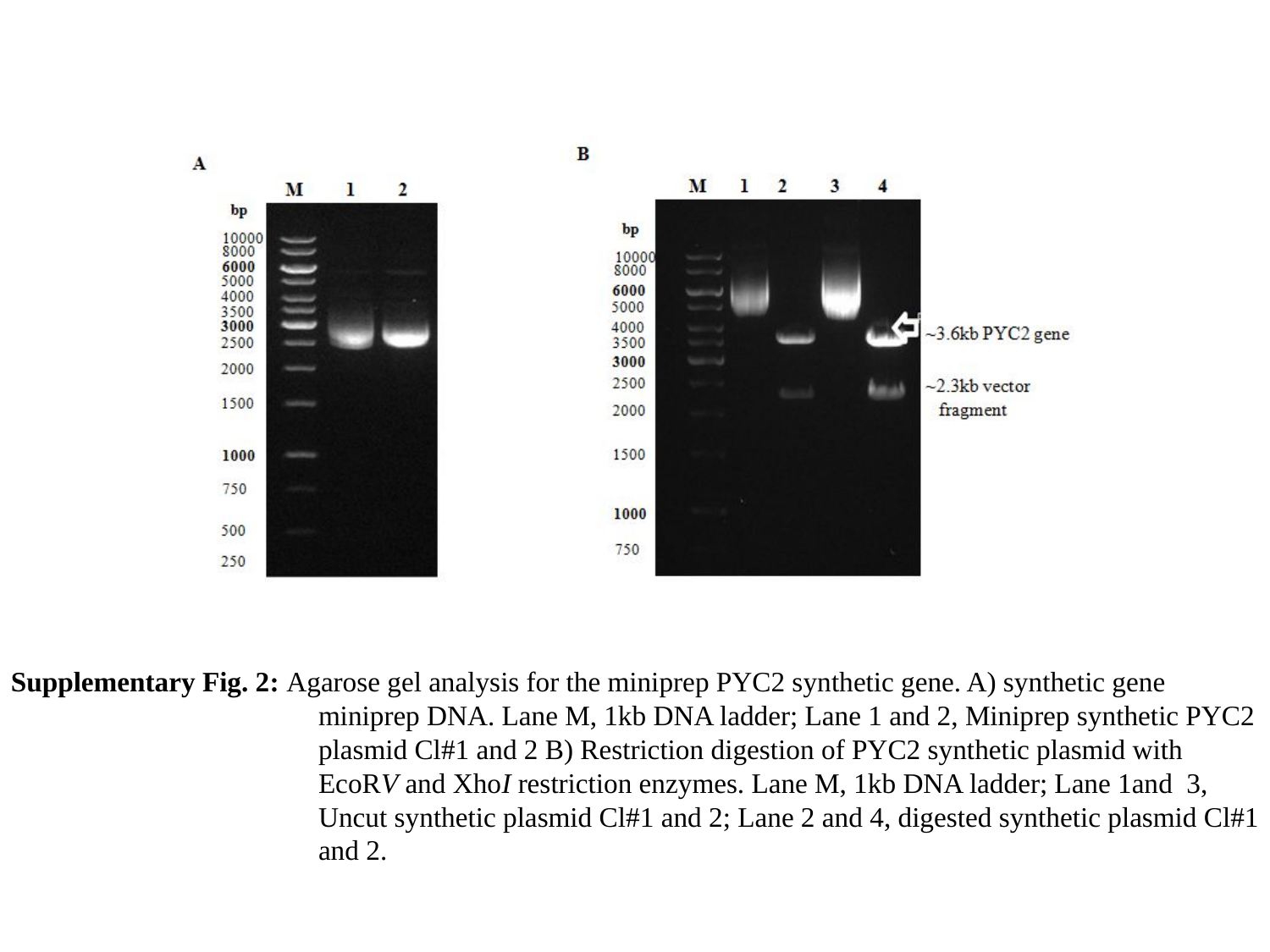

Supplementary Fig. 2: Agarose gel analysis for the miniprep PYC2 synthetic gene. A) synthetic gene
 miniprep DNA. Lane M, 1kb DNA ladder; Lane 1 and 2, Miniprep synthetic PYC2
 plasmid Cl#1 and 2 B) Restriction digestion of PYC2 synthetic plasmid with
 EcoRV and XhoI restriction enzymes. Lane M, 1kb DNA ladder; Lane 1and 3,
 Uncut synthetic plasmid Cl#1 and 2; Lane 2 and 4, digested synthetic plasmid Cl#1
 and 2.
